# Use of procalcitonin in therapeutic decisions in the pediatric intensive care unit

**DOI:** 10.1186/s13613-025-01470-y

**Published:** 2025-04-23

**Authors:** Pierre Tissières, Elisabeth Esteban Torné, Johannes Hübner, Adrienne G. Randolph, Corsino Rey Galán, Scott L. Weiss

**Affiliations:** 1 IHU-PROMETHEUS Comprehensive Sepsis Center, Pediatric Intensive Care, Neonatal Medicine and Pediatric Emergency Department, AP-HP Paris Saclay University, Bicêtre Hospital, 78, Rue du General Leclerc, 94275 Le Kremlin-Bicêtre, France; 2 University of Barcelona, Hospital Sant Joan de Déu, Barcelona, Spain; 3 Ludwig-Maximilian-University, Hauner Children’s Hospital, Munich, Germany; 4 Department of Anesthesiology, Critical Care and Pain Medicine, Boston Children’s Hospital, Boston, MA USA; 5 University of Oviedo, Hospital Universitario Central de Asturias (HUCA), Health Research Institute of the Principality of Asturias (ISPA), Oviedo, Spain; 6 Thomas Jefferson University, Nemours Children’s Health, Jacksonville, DE USA

**Keywords:** Procalcitonin, Sepsis, Bacterial infections, Pediatric intensive care units

## Abstract

Procalcitonin (PCT) is frequently used by clinicians in children with suspected bacterial infections and sepsis. However interpretation in the critically ill child may be challenging due to the complexity of underlying conditions and its impact on PCT values. Herein, we propose a guidance for the use of procalcitonin in critically ill children, supported by a comprehensive analysis of the literature, to help the clinician for interpreting PCT in the various clinical conditions encountered in pediatric intensive care units. We describe the importance of the clinical context, timing of measurement and evidence on PCT values in diagnosing sepsis and to guide antibiotic therapy in critically ill children.

**Graphical abstract:**

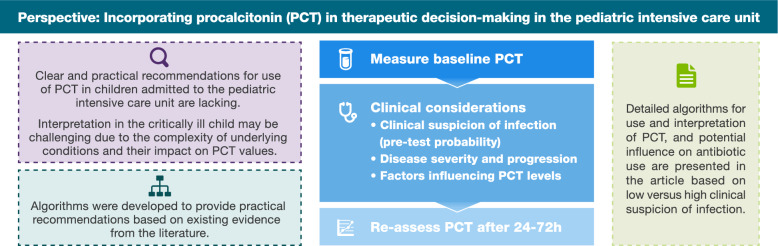

## Background

Sepsis remains one of the principal threats in critically ill children admitted to the pediatric intensive care unit (PICU) where timely and appropriate antimicrobial therapy is the standard of care to optimize health outcomes [1]. However, unnecessary antibiotic increases risk of adverse effects and promotes antimicrobial resistant pathogens without patient benefits. Antibiotic stewardship programs have been shown to effectively reduce antibiotic overuse in PICU patients without influencing mortality or morbidity [2].

Procalcitonin (PCT) is a biomarker that is often used by clinicians in children with suspected bacterial infections and sepsis [3, 4]. However, optimal use of PCT in the pediatric intensive care unit (PICU) is limited by the lack of clear and practical recommendations based on existing evidence [5]. Furthermore, appropriate interpretation of PCT levels depends on the clinical context and the evolution of illness severity. Indeed, in certain clinical situations encountered in PICU, PCT levels can be high and remain high in the absence of a bacterial infection or sepsis. In contrast, in some cases, PCT levels can be low and remain low despite the presence of infection requiring antibiotic therapy [6]. The most recent pediatric Surviving Sepsis Campaign (SSC) guidelines do not evaluate the use of PCT for children [7].

In this perspective, we present an expert review of the evidence on how to use PCT to assist with diagnosis of bacterial infection and guide therapeutic management of acute and critically ill children and provide practical recommendations based on existing literature.

### How to interpret procalcitonin levels for antibiotic stewardship in acute and critically ill children ?

To appropriately incorporate PCT levels into clinical decision-making for acute and critically ill children, it is necessary to understand the pharmacokinetics and pharmacodynamics of PCT [7]. Typically, PCT is predominantly produced by thyroid C-cells and then rapidly converted to calcitonin as part of calcium homeostasis [3, 8, 9]. Thus, under normal physiological conditions PCT levels in the blood remain extremely low. During systemic bacterial infections, expression of the calcitonin 1 gene (CALC-1) is upregulated in nearly all tissues in response to elevated levels of interleukin (IL)-6, tumor necrosis factor (TNF)-α, and IL-1β. However, outside of the thyroid and some neuroendocrine tissues, intracellular conversion of PCT to calcitonin does not occur [3, 8]. Thus, during systemic bacterial infections, PCT transits into the blood with measurable increases in circulating PCT levels. With onset of a systemic bacterial infection, PCT can usually be detected in the blood after 3–4 h and peaks within 6–48 h [3, 8–10]. The precise timing and peak value of circulating PCT in bacterial infections depends on the pathogen and the focus of infection [11]. In contrast, PCT levels often remain low in the blood during localized bacterial infections in which a systemic cytokine response is minimal, even in cases of severe local infections (e.g., some cases of endocarditis and osteomyelitis) [8]. During viral infections, the increased secretion of interferon-γ, attenuates the upregulation of CALC-1, inhibiting the production of PCT and keeping serum PCT levels relatively low [8]. The half-life of PCT is between 22 and 35 h. Thus, after appropriate source control and successful therapeutic management, blood PCT levels decrease and return to normal within a few days [8–12]. Although the purpose of this review was not to compare PCT to other available biomarkers such as CRP, the combination of fast kinetics and specificity for bacterial infections make PCT an optimal currently available biomarker for assessing the risk for a bacterial infection and for successful antibiotic therapy monitoring.

#### Clinical situations with elevated procalcitonin levels without bacterial infection

Blood PCT levels can be elevated in patients with severe systemic inflammation from non-bacterial causes, including respiratory failure caused by severe viral respiratory infections [13, 14], malaria, bowel wall ischemia, vasculitis or cancer [3]. PCT levels also increase after surgery [3, 15–17], burns [3, 18], trauma [6], shock [19], cardiac arrest [19] and in patients with invasive candidiasis [20]. For example, a retrospective study of 646 children with PCT levels measured within 48 h of being admitted to the PICU [21] identified 33 patients with elevated PCT levels despite no evidence of systemic bacterial infections. Among these, 25 (76%) were patients admitted after either surgery, trauma, cardiac arrest, immunomodulatory therapy, or with acute kidney injury or dehydration. In those patients, following resolution of inflammation, PCT levels gradually decrease during the following 3–4 days. In neonates, there is a natural physiological increase in PCT levels after birth through 24–72 h which, in the absence of a bacterial infection, also gradually decreases within a few days [3, 22].

Patients with acute [23–25] or chronic kidney injury [23, 24] can have sustained elevation of blood PCT levels. However, in these patients a bacterial infection will trigger a further elevation in PCT compared to baseline. During continuous renal replacement therapy (CRRT), blood PCT levels usually decrease when there is no infection, or when the infection is controlled [26]. An increase or a lack of decrease in PCT levels within 24–48 h after starting CRRT may indicate that the source of the infection has not been adequately controlled [26, 27].

#### Clinical situations of bacterial infections without elevated procalcitonin levels

Depending on the timing of the PCT measurement and/or the type of infection, PCT levels may not be elevated in the blood even in the presence of a bacterial infection. If the measurement of PCT is performed very early during an infection, the PCT level may not yet be sufficiently elevated to provide additional evidence of a bacterial infection. Furthermore, localized infections (e.g., surgical wound infection, abscess, osteomyelitis, endocarditis, bacterial pericarditis, ventriculitis/ventriculoperitoneal shunt infection), even if severe, may not result in an increase in the circulating PCT levels. Therefore, correct interpretation always requires considering potential sites of infections and its related impact on PCT measurements. As an example, a retrospective study performed in children admitted to the PICU who were not in shock identified 17 patients with low PCT levels despite confirmation of localized bacterial infections [21]. Among these, 10 patients (59%) had either a surgical site infection, a bone infection, infective pericarditis, retropharyngeal abscess, and ventriculitis/ventriculoperitoneal shunt infection.

### What are the clinical evidences for the use of procalcitonin?

Current evidence for the use of PCT in acute and critically ill children admitted to PICU is limited. However, there is some evidence that PCT levels can be used when bacterial infections are suspected in hospitalized children (not in the PICU) [28–30]. PCT levels have also been used to improve antibiotic stewardship by identifying patients at very low risk of bacterial infections [31–34].

#### Procalcitonin levels in hospitalized patients

Use of PCT levels to assist clinicians with antibiotic stewardship has been applied in children with lower respiratory tract infection, including community-acquired pneumonia (CAP), by identifying those with very low likelihood that the infection is bacterial [5, 28–30]. Initially, antibiotics are essential in children with clinical suspicion of bacterial CAP [35]. In intubated patients, delaying antibiotics within the first two days, increases the duration of hospitalization and time on mechanical ventilation [36]. However, the etiology of CAP is proven to be bacterial in only about 15% of hospitalized children [37]. In most children, CAP is viral, and antibiotics are not of benefit. In the Etiology of Pneumonia in the Community (EPIC) study of 532 US children hospitalized with radiographically confirmed CAP, none of the 52 children with a typical bacterial infection (10.2%) had a PCT level below 0.1 µg/L (equivalent to measure in ng/mL) [30]. For PCT levels below 0.25 µg/L, the negative predictive value for excluding a typical bacterial infection was 96% (95% confidence interval [CI]: 93–99%), while sensitivity was 85% (95% CI 76–95%), and specificity was 45% (95% CI 40–50%) [30]. Thus, hospitalized children with CAP who had a PCT below 0.25 µg/L had a fivefold lower likelihood of having an infection caused by a typical bacterial organism, helping to identify patients with a low risk of a typical bacterial etiology for whom it would likely have been safe to withhold antibiotics. Although a very low PCT of less than 0.1 µg/L increases the certainty that the infection is not bacterial, using this cutoff lowers the positive predictive value for the great majority of patients with PCT values between 0.1 and less than 0.25 µg/L who do not have a bacterial infection. It is noteworthy that in the EPIC study, PCT levels were also low in the 82 (15.4%) children with bacterial infections caused by atypical organisms (*Mycoplasma pneumoniae* or *Chlamydophila pneumoniae*), who may also benefit from antibiotics [30]. In contrast, elevated PCT above 0.25 µg/L is common among children requiring non-invasive or invasive mechanical ventilation due to viral illnesses (e.g., bronchiolitis) [21], making it less useful in confirming that an infection is bacterial.

#### Procalcitonin levels in immunocompromised patients

In immunocompromised children, the inflammatory response to bacterial pathogens may be severely impaired with a high risk of severe complications from infections. In this setting the use of PCT, and other biomarkers of infection, warrant specific guidelines. The literature suggests that PCT may be better than CRP in diagnosing bacteremia in immunocompromised children (e.g., those with cancer or after hematopoietic stem cell transplantation) [38, 39]. However, the diagnostic accuracy of PCT for sepsis is limited in this population. In this setting, a higher rate of false negative PCT measurements can be expected, and physicians must systematically investigate possible local infections. Following hematopoietic stem cell transplantation (in adults) and fever during neutropenia in children with cancer, a PCT value higher than 0.25–0.5 µg/L suggests systemic bacterial or fungal infections, with values below 1.0 µg/L associated with severe infections and mortality [40, 41]. Serial measures of PCT, in children with low-risk febrile neutropenia receiving chemotherapy, may help reduce the duration of antibiotics [42].

#### Procalcitonin levels after surgery, trauma, and cardiac shock

Non-infectious inflammatory triggers, such as surgery, trauma, and shock can increase PCT levels to an extent similar to that observed with bacterial pathogens [43–45]. However, the distinct kinetics of PCT in these clinical scenarios can be helpful at the bedside. In children following surgery, trauma, and cardiac shock, the increase in PCT is transient, peaking within 48 h, with the exact time of peak values dependent on the type of surgery [46]. PCT levels then gradually and predictably decrease in the absence of an infection. In contrast, for patients with a concurrent or new bacterial infection, PCT will continue to increase if the source of infection is not sufficiently controlled. PCT levels that do not decrease or that continue to increase for more than 1–2 days following surgery, trauma, or non-septic shock are associated with adverse outcomes [46]. In these patients, a possible bacterial infection should be investigated. Studies have assessed the benefit of measuring PCT levels in patients after surgery [47, 48], burns [49, 50], and cardiac arrest [51]. A systematic review evaluated PCT levels in children after cardiac surgery found that PCT levels peak 2 days after surgery [47]. The authors suggest that in this setting, infection should be suspected when PCT levels exceed 5.6 µg/L within 48 h of surgery.

A meta-analysis in patients (adults and children) with burns found that sepsis should be suspected in patients with PCT levels above 1.5 µg/L [4, 52]. A retrospective study reported that PCT levels were significantly higher in patients with sepsis within the first 5 days after a burn injury, with PCT levels higher than 1.0 µg/L associated with sepsis [50]. In children after cardiac arrest, a systematic review and meta-analysis assessing PCT levels concluded that elevated levels within the first 48 h were associated with poor outcomes [51]. Although these studies suggest cutoffs for PCT levels, these cutoffs were obtained for different conditions and have not been clearly established. Further data to confirm the clinical utility of PCT cutoffs after pediatric cardiac arrest is required. However, in these situations there is a benefit in sequential measurements of PCT to understand the kinetics. These results, together with other biomarkers and clinical assessments, may be useful for managing children after surgery, burns, and cardiac arrest.

#### Procalcitonin measurements and antibiotic stewardship

A systematic review and cost-effectiveness analysis assessed the potential benefit of using PCT for antibiotic stewardship during the treatment of sepsis and suspected bacterial infections in adults and children [53]. The study found that PCT may be useful in two situations: (1) to guide safe discontinuation of antibiotics in adults with suspected or confirmed sepsis and (2) to guide initiation of antibiotics in adults presenting with respiratory symptoms with possible bacterial infection. More recently, two controlled trials evaluating efficacy of a PCT-based algorithm to reduce antibiotic therapy duration showed diverging results. The ADAPT study confirm a reduced duration of antibiotic therapy in critically-ill adults from 10.7 to 9.8 days [54]. In contrast, the BATCH study did not demonstrate an effect on reducing antibiotic therapy duration compared to usual care without PCT (4.0 vs. 4.1 days) in a general pediatric population with few critically ill children [55]. Although in the BATCH study the algorithm appeared to be safe, the trial had limitations including methodological limits in the use of PCT value for decision making, poor adherence to the PCT algorithm and implementation of antibiotics stewardship programs before the study started [55].

Studies in children with CAP [56, 57], hospital-acquired infections [33], severe bronchiolitis [34], and after cardiovascular surgery [32] have shown that PCT levels can be used to safely reduce overall antibiotic use in children. Three randomized controlled trials have assessed PCT guided algorithms or cutoffs for antibiotic stewardship [56–58]. A randomized clinical trial assessed whether a PCT cutoff of 0.25 µg/L could be used to guide antibiotic therapy in 319 children hospitalized with uncomplicated CAP [56]. In the experimental group, children with admission PCT levels below 0.25 µg/L were not treated with antibiotics. Those with PCTs levels higher or equal to 0.25 µg/L were treated with antibiotics until their PCT levels decreased below this established threshold. Compared to the control group, fewer patients in the PCT guided group were prescribed antibiotics, the duration of antibiotic use was shorter, and there were fewer antibiotic-related adverse events, while the recurrence of respiratory symptoms and new antibiotic prescription in the month following enrollment were unchanged. It is important to note that only children with mild to moderate CAP were assessed in the trial; therefore, the use of this algorithm in more severely ill patients was not tested. A similar randomized trial that enrolled 337 children with lower respiratory tract infections allocated antibiotics either according to a PCT guided algorithm or usual care [56]. The PCT algorithm recommended that children with PCT levels above 0.5 mg/L be “definitely treated” with antibiotics and those with PCT levels below 0.1 mg/L “definitely not be treated” with antibiotics. The algorithm could have been overruled by physicians for children with life threatening infections. While the use of the algorithm did not reduce the initial antibiotic prescription rate, PCT guidance did reduce overall exposure to any antibiotics and the duration of antibiotics (from 6.3 days in the standard of care group to 4.5 days with the algorithm). A combined safety endpoint, which included, amongst others, complications from LRTI, hospital readmission, and severe adverse events, showed no difference between the groups. Recently, a randomized, blinded, clinical trial assessed the benefit of using PCT and lung ultrasound (LUS) compared to chest radiography (CXR) to manage children with bacterial pneumonia [57]. Critically ill children with suspected hospital-acquired pneumonia or CAP allocated to the control group (n = 98) had a CXR and those allocated to the experimental group (n = 96) had a LUS. PCT determinations were performed in all patients. The decision to prescribe antibiotics was based on imaging test results and PCT levels. If the CXR or LUS did not suggest bacterial pneumonia and PCT levels were below 1.0 µg/L, then antibiotics were not prescribed. If the CXR or LUS suggested bacterial pneumonia, antibiotics were recommended regardless of the PCT level. If the CXR or LUS did not suggest a bacterial pneumonia, but PCT levels were higher than 1.0 µg/L, antibiotics were also recommended. This study found LUS combined with PCT levels to be more sensitive and specific for diagnosing bacterial pneumonia (sensitivity was 90% and specificity was 85%) compared to LUS or CXR alone, and CXR combined with PCT levels. A secondary analysis showed that the LUS-PCT combined algorithm could reduce the radiation exposure without an increase in costs and without risk of mistreating bacterial pneumonia [59].

### Could procalcitonin be helpful for guiding antibiotic initiation?

The 2021 SSC adult guidelines do not support the use of PCT levels to initiate antimicrobials [60, 61]. These recommendation were based on the results from a meta-analysis that reported that a PCT cutoff of 1.1 µg/L could distinguish patients with and without sepsis with a sensitivity of only 77% and specificity of 79% [62]. The SSC expert committee considered that the sensitivity and specificity of PCT was not sufficient in high-risk situations like sepsis. Moreover, the reported inter-patient variability in PCT levels is too large to establish fixed cutoff levels [63, 64]. The SSC guidelines do support the use of PCT with clinical data to discontinue antimicrobials in adults, but do not provide guidance on how this should be done [54, 55]. The 2020 SSC guidelines for pediatrics [1] do not comment on or provide guidance for the use of PCT. Similarly, no recommendation concerning the use of PCT are provided in the NICE sepsis guidelines for adults or children (in the United Kingdom) [65]. However, they did publish an evidence-based review of PCT in sepsis [66]. They concluded that the use of PCT is promising, but that the current level of evidence is not sufficient to include PCT in routine practice. The NICE guideline for suspected meningitis and meningococcal disease in children recommends performing PCT as an alternative if CRP is not available, as part of a panel of initial tests, and notes that a low PCT does not rule these infections out [67]. The American Thoracic Society (ATS) guideline for CAP recommend that PCT levels be used to distinguish upper from lower respiratory tract infections in adults [68]. However, the ATS does not recommend using PCT to decide whether antibacterial therapy should be initiated. Empiric antibiotic therapy should be given to adults with clinically suspected and radiographically confirmed CAP regardless of the initial blood PCT level. The reasons for the non-support of using PCT levels were due to the large variability in PCT levels depending on the specific bacterial pathogen [69] and the variable specificity and sensitivity observed with PCT cutoffs [70].

In children, the American Academy of Pediatrics (AAP), in their recent guideline for the evaluation and management of well-appearing febrile infants aged 22–60 days, included a PCT cutoff of 0.5 µg/L in their evaluation and management algorithms to suggest further diagnostic investigations and consideration of antimicrobial therapy while awaiting the results of blood cultures [71].

### Could procalcitonin be helpful for guiding antibiotic continuation once started?

In critically ill children, PCT must be combined with a thorough clinical evaluation and interpreted along with other inflammatory and organ-specific biomarkers [72]. To cover most clinical conditions encountered in PICU, we suggest measuring PCT at the time of infection suspicion and systematically repeating its measurement within 24–72 h. In patients with low clinical suspicion of infection, an initial PCT value below 0.25 μg/L in combination with negative microbiological workup, repeated PCT measures are probably unnecessary. In patients with an increasing PCT trend, unchanged or worsening clinical conditions, it is advisable to consider alternative diagnoses, other or profound foci of infection, and/or broaden antibiotic therapy. When interpreting PCT levels in critically ill children, it is essential to consider clinical situations where PCT can be elevated in the absence of bacterial infections and can remain low despite the presence of bacterial infections. Given the higher risk of severe outcomes from infections in immunocompromised children and insufficient data on the use of PCT in these patients, guidance should consider whether to include this population in the recommendations.

It is noteworthy that the introduction of PCT use in the PICU without an accompanying algorithm guidance or stewardship intervention found no change in antibiotic use before or after introduction of PCT testing [73]. In contrast, when practical algorithms that included PCT levels were integrated into clinical decision-making, benefits were observed [57, 74, 75]. Most of the current evidences support the use of PCT-guided algorithms to avoid unnecessary antibiotics when clinical risk of bacterial infection is low or to discontinue antibiotics when there is clinical improvement, no indication of a bacterial infection, and/or when PCT levels remain low, decrease by 80% of their maximum values, or fall below 0.5 µg/L. When added to standard clinical care, one study showed that early measurement of PCT can help to identify children admitted to the PICU with signs and symptoms of potential infection who are at low risk of bacterial infection [76]. In patients without confirmatory evidence of bacterial infection at 48 h, a PCT level below 1.0 µg/L (in conjunction with a CRP level below 4 mg/dL) could be safely used to exclude the notion that clinical improvement was attributable to antibiotic treatment of an unidentified bacterial infection such that it would be appropriate to discontinue further antibiotics. Currently, there is not enough evidence in children to suggest that PCT levels should be used for antibiotic escalation or to shorten the duration of a full antibiotic course in those with a confirmed bacterial infection. However, in children with a high clinical suspicion of bacterial infection or sepsis, with a clinical course not improving or worsening, further investigations and potential broadening of antibiotic therapy may be warranted according to the physician’s interpretation of all of the clinical evidence.

#### Perspectives and conclusion

Considering the current evidence, two algorithms were compiled based on the clinical suspicion of a bacterial infection and disease severity of children admitted to the PICU. Two populations of PICU patients were considered, based on whether the clinician has a high or low level of clinical suspicion of bacterial infection. Notably, the algorithms that we propose here are similar to the approach used in the recent BATCH study in which a PCT-guided algorithm for antibiotic duration was non-inferior to usual care without PCT among all patients, but did safely reduce the median antibiotic duration in the subset with adherence to the PCT-guided algorithms. Based on the data presented above and these recent findings from the BATCH study, it is likely that PCT will remain in common use [55]. Thus, we offer these algorithms to guide the application of PCT for children admitted to the PICU.

#### Low clinical suspicion of bacterial infection

**Fig. 1:**
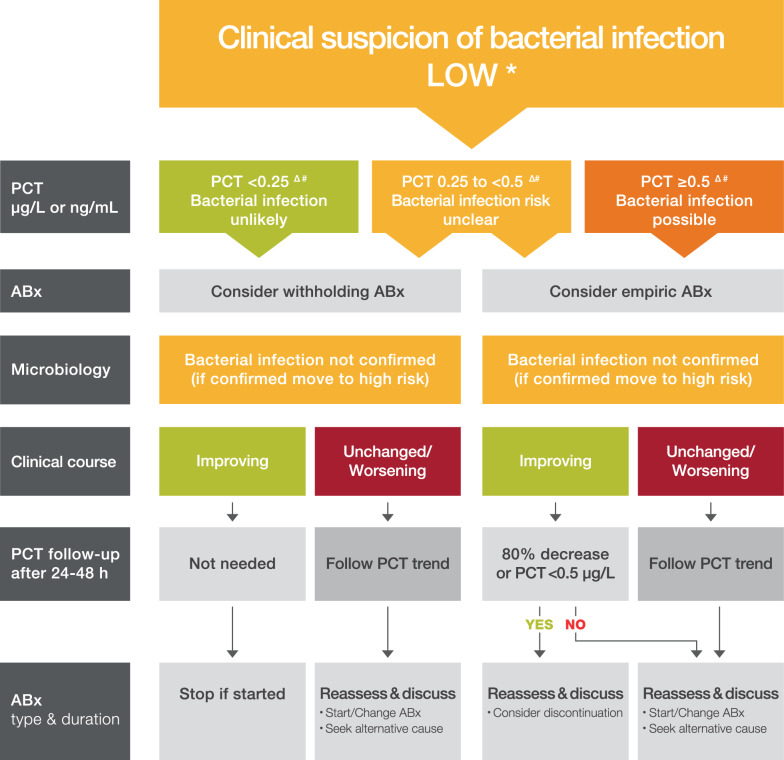
Algorithm for incorporating procalcitonin in therapeutic decision-making in patients with an initially low clinical suspicion of bacterial infection based upon medical history and clinical condition. Low suspicion of bacterial infection without signs of sepsis, shock or organ dysfunction. *Caution in patients with immunosuppression (including HIV), CF, pancreatitis, trauma, high volume transfusion, malaria, patients undergoing ECMO, CART-T therapy. The algorithm should not be applied in patients with chronic infections (e.g., abscess, osteomyelitis, endocarditis) or in neonates. ^Δ^PCT may be elevated due to non-infectious cause post-operative, post-cardiac arrest, heat stroke, burns trauma renal failure. ^#^PCT may be low despite an infection due to an early course of the infection, focal infections, fungal infection or therapy (steroids, CRRT, antimicrobials…). Abx, antibiotics; PCT, procalcitonin

In patients with an initially low clinical suspicion of bacterial infection (Fig. 1) based upon medical history and clinical condition, where the microbiological workup subsequently confirms that bacterial infection is unlikely, PCT levels that are initially low or decreasing over time can help confirm the absence of a bacterial infection and allow for ongoing treatment without antibiotics or safe discontinuation of antibiotics if started. However, if PCT levels are rising or constantly high, further workup to investigate for a possible bacterial infection is recommended. We chose a conservative PCT level of < 0.25 µg/L as the cutoff based on existing literature in hospitalized children and lack of data to justify a higher cutoff. However, it is likely that this cutoff may be highly sensitive but lacks specificity in the PICU population, and further research is recommended. An additional range for PCT levels between 0.25 and 0.5 µg/L was added where PCT needs to be interpreted, with all other clinical and laboratory information. Note that if concurrent evaluations support a localized bacterial infection (e.g., positive urinalysis that suggests presence of a lower urinary tract infection; cerebrospinal fluid pleocytosis in a patient with fever and ventriculoperitoneal shunt) treatment with appropriate antibiotics is warranted irrespective of the PCT level.

#### High suspicion of bacterial infection

**Fig. 2:**
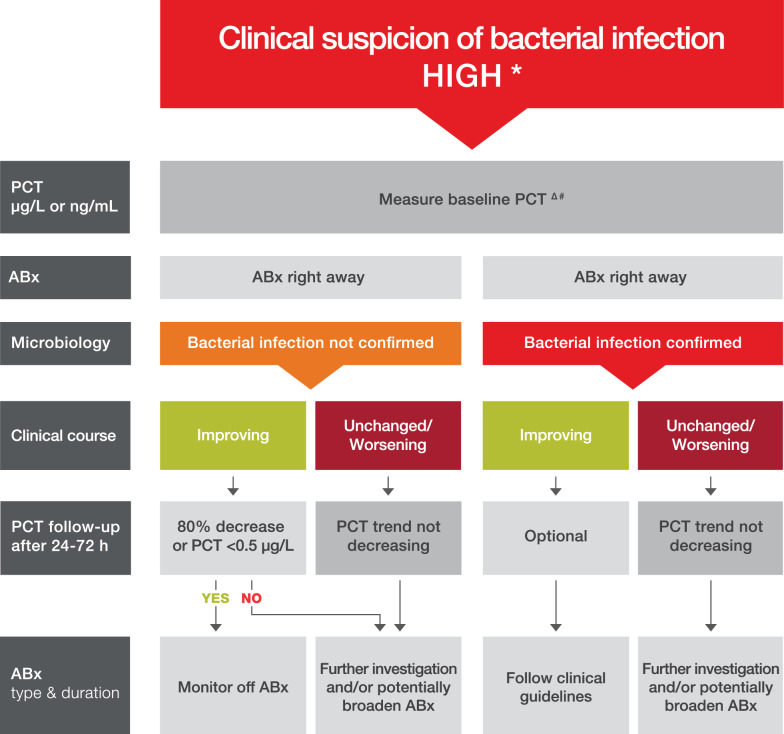
Algorithm for incorporating procalcitonin in therapeutic decision-making in patients with a high suspicion of a bacterial infection. High suspicion of bacterial infection such as clinical signs of shock, organ dysfunction or other evidence of bacterial infection (e.g., empyema). *Caution in patients with immunosuppression (including HIV), CF, pancreatitis, trauma, high volume transfusion, malaria, patients undergoing ECMO, CART-T therapy. The algorithm should not be applied in patients with chronic infections (e.g., abscess, osteomyelitis, endocarditis) or in neonates. ^Δ^PCT may be elevated due to non-infectious cause post-operative, post-cardiac arrest, heat stroke, burns trauma renal failure. ^#^PCT may be low despite an infection due to an early course of the infection, focal infections, fungal infection or therapy (steroids, CRRT, antimicrobials…) Abx, antibiotics; PCT, procalcitonin

For children with a high suspicion of a bacterial infection (Fig. 2), such as clinical signs of sepsis/septic shock, organ dysfunction, or other evidence of bacterial infection (e.g., empyema), antibiotics should be initiated without necessarily considering the initial PCT value. However, measuring PCT at this early timepoint can still be beneficial as a starting point to trend PCT over time to evaluate response to therapy depending on the outcome of the microbiology and the patient’s clinical course. Data are lacking in children regarding the optimal absolute PCT value or percentage decrease that indicates that the response to therapy is appropriate, and further research is recommended.

In summary, we present a decision-making approach for using PCT in the PICU. The use of PCT in critically ill children should not be limited to the hours after admission but should extend throughout the entire PICU hospitalization. The two algorithms covering low- and high-suspicion bacterial infections may address most clinical conditions encountered in the PICU and serve for a future controlled trials in critically-ill children. Although additional data are needed for critically ill children, this narrative review demonstrates the importance of published experience using PCT in critical care and its strong potential for infection diagnosis and sepsis management, especially when considering the clinical conditions that may influence the correct interpretation of PCT measurements in critically ill children.

## Data Availability

The data used in this narrative review have already been published.

## Abbreviations

Abx: Antibiotics
HIV: Human immunodeficiency virus
ECMO: Extracorporeal membrane oxygenation
CAR-T: Chimeric antigen receptor T cells
PCT: Procalcitonin
CF: Cystic fibrosis
